# Olfactory gene dynamics in invasive Indian and non-invasive African malaria vectors at the crossroads of development, infection and resistance

**DOI:** 10.1038/s41598-025-21404-9

**Published:** 2025-10-28

**Authors:** Arvind Sharma, Bhuvan Dixit, Bharti Goyal, Renuka Harit, Jatin Kumar, Shibani Biswas, Ritu Goswami, Kailash C. Pandey, S. Noushin Emami, Soumyananda Chakraborti

**Affiliations:** 1 ICMR-National Institute of Malaria Research, Dwarka, New Delhi, India; 2 Department of Biochemistry and Molecular Biology, University of Nevada, Reno, USA; 3 Academy of Scientific and Innovative Research (AcSIR), Ghaziabad, UP India; 4 Natural Research Institute, University of Greenwich, Greenwich, UK; 5 Department of Vector Biology, Liverpool School of Tropical Medicine, Liverpool, L3 5QA UK; 6 Molecular Attraction AB, Elektravägen 10, Hägersten, 126 30 Stockholm, Sweden; 7 Department of Microbiology, Tumor and Cell Biology, Karolinska Institute, Stockholm, Sweden; 8 Department of Biological Sciences, BITS-Pilani (Hyderbad), Hyderabad, India

**Keywords:** Indian invasive malarial vector, African noninvasive vector, Mosquito olfaction, Insecticide resistance, Synteny, *CSP* and *SAP* expression in mosquito larvae, Cell biology, Microbiology, Molecular biology, Structural biology, Ecology

## Abstract

Olfaction plays a pivotal role in a mosquito’s lifecycle, influencing vital functions such as finding food, mates, identifying hosts, and locating sites for laying eggs. However, a detailed catalog of the olfactory genes in mosquitoes has remained elusive—until now. In this study, we compiled the olfactory genes catalog for four key malaria vectors: two major Indian species, *Anopheles stephensi* and *Anopheles culicifacies*, along with two African species, *Anopheles gambiae* and *Anopheles funestus*. Using an extensive genome-wide approach, we uncovered crucial carrier proteins like odorant binding proteins (*OBPs*), chemosensory proteins (*CSPs*), and several receptors, including odorant receptors (*ORs*), ionotropic receptors (*IRs*), and gustatory receptors (*GusRs*). A particularly striking discovery was the significantly higher number of *OBP*, *OR*, and *IR* genes in African malaria vectors compared to their Indian counterparts, hinting at the gene gain and functional diversification in these species. The invasive *A. stephensi*—which has spread from Asia to Africa—showed closer genetic ties to *A. minimus* and *A. gambiae* than to *A. culicifacies*. Furthermore, when examining the expression of *CSPs* and *SAPs* in the larval stage of *A. stephensi*, we found that pyrethroid-resistant mosquito larvae exhibited elevated expression of *SAP2* and *SAP3*, providing new evidence of their potential role in insecticide resistance. This study not only sheds light on the genetic basis of mosquito olfaction but also offers crucial insights into how these genes are linked to different physiological functions, paving the way for improved malaria control strategies.

**Supplementary Information:**

The online version contains supplementary material available at 10.1038/s41598-025-21404-9.

## Introduction

Mosquitoes belonging to the Anopheline (*Anopheles*) subfamily are part of a large Diptera: Culicidae family found across the inhabited world^1^. Adult females rely on blood meals, predominantly from vertebrate hosts such as birds, frogs, mammals, and snakes, to complete their egg development^2,3^. Among these hosts, humans have the unfortunate distinction of being a preferred blood meal source by certain *Anopheles* species which can lead to the transmission of parasites harbored by these mosquitos^4,5^. *Anopheles* mosquitoes, in particular, are recognized as highly perilous hematophagous arthropods due to their ability to transmit the malaria-causing *Plasmodium* parasites. Tragically, malaria causes the loss of more than 0.4 million lives each year, inflicting a catastrophic toll on a global scale. World Health Organization (WHO) Report 2024 clearly shows that there has been a steady decline in global malaria deaths between 2000 and 2019. However, in 2023, the number of malaria-related deaths rose to 0.597 million, accompanied by an increase of 11 million new cases. The incidence rate also climbed, rising from 58.6 to 60.2 cases per 1,000 individuals at risk compared to 2022^6^.

Their coexistence ensures sustained transmission throughout the year, posing challenges for malaria control. During the rainy season, when there is an abundance of water sources, *A. gambiae* population thrives. These mosquitoes prefer breeding in temporary rain-dependent pools and show a peak in their activity during this period. Just after rainy season dry season arrives and the occurrence of *A. gambiae* declines, and *A. funestus* replaces *A. gambiae* as the predominant mosquito species involved in malaria transmission^7,8^. In India, the two most dominant *Anopheles* species, *A. culicifacies* and *A. stephensi* act as significant malaria vectors in rural and urban settings and are responsible for the majority of malaria cases^8,9^. *A. stephensi* is an invasive malaria vector that is originally endemic in Asia and competent in transmitting both malaria parasites *Plasmodium falciparum* and *Plasmodium vivax*^9,10^. There is increasing evidence that *A. stephensi* is expanding its geographical range, with the type form being found in Sri Lanka in 2016^10–12‚11‚12‚13^ and crossing from the Arabian Peninsula into Africa where it has been reported in Djibouti City in 2012, in Ethiopia in 2016 and 2018^13‚14^ and in 2019, it was detected in the coastal and sub-coastal regions of the Red Sea in Sudan^14‚15^. *A. stephensi* differs from other primary malaria vectors in its remarkable ability to survive and reproduce in urban environments^15‚16^. This species has a high degree of ecological plasticity, enabling it to adapt quickly to diverse local environments such as deep wells under extreme heat in the dry season and is capable of breeding in saline water as well as polluted water^16‚17^. Therefore, the emergence of this highly adaptable super mosquito can drive a new malaria surge across Africa, as the arrival of this a city-dwelling mosquito from Asia presents a serious risk to rapidly urbanizing Africa. As a result of this, WHO has raised an alarm about the invasion and spread of *A. stephensi* into Africa. The establishment of *A. stephensi* in Africa poses a potential threat to malaria control and elimination, which further has a global importance and impact.

Mosquitoes heavily rely on their sensory abilities to carry out vital tasks for their survival and reproductive success. One crucial sense they rely on is their sense of smell, or olfaction, which plays a critical role in various aspects of their life cycle. For instance, when it comes to obtaining food from plants (nectar), mosquitoes use olfaction to detect and locate sources of sugar-rich plant juices^17‚18^. The sense of smell is also essential for mosquitoes to find suitable mates. Male mosquitoes rely on their olfactory receptors to sense pheromones, which empowers them to navigate scent trails, gather with other males to form swarms, and seek out potential mates for reproduction^18‚19^. When it comes to blood feeding, olfaction is again crucial as female mosquitoes rely on its sensitive and efficient sensory system to find vertebrate hosts^2^. Additionally, mosquitoes are attracted to carbon dioxide (CO_2_) that humans and other animals emit during respiration. They have the ability to sense the concentration of CO_2_ in the air, which aids them in locating potential hosts for blood meals. Furthermore, olfaction plays a vital role in oviposition, or the process of female mosquitoes laying their eggs. They can sense chemical cues from water sources, which indicate the availability of suitable breeding sites for their offspring. These cues may originate from microorganisms or organic matter, such as decaying leaves, signaling the presence of nutrients necessary for the development and survival of mosquito larvae^17‚18^.

Mosquitoes possess three important structures dedicated to their sense of smell: the antenna, maxillary palps, and labella. These appendages house specialized receptors that enable them to detect odors within their chemical surroundings. Among these structures, the mosquito antenna takes on the crucial role of being the primary organ responsible for smelling. Within the intricate olfactory system of mosquitoes, three primary categories of receptors play significant roles: odorant receptors (*ORs*), ionotropic receptors (*IRs*), and gustatory receptors (*GusRs*). These receptors are expressed by the olfactory neurons present in the sensory hairs, known as sensilla, located on the olfactory appendages^17,19‚18‚20^. It’s important to note that each of these receptor classes comprises multiple subunits, collectively forming a functional ion-gated channel. OR consists of a tuning receptor (ORx) and a conserved co-receptor (Orco)^20‚21^. The IR consists of a tuning receptor (IRx) which confers substrate (ligand) specificity on the neuron, in addition to one or more coreceptors (Ir8a, Ir25a, and/or Ir76b) which are highly conserved. GusR complexes consist of 3 subunits (Gr22, Gr23, and Gr24) which together sense CO_2_ and sugars^21,22‚23^. On the other hand, ORs are sensitive to compounds like esters, alcohols, and ketones, while OBPs respond to various amines and acids^23‚24^. Besides these three classes of receptors, there are some water-soluble accessory proteins such as chemosensory proteins (CSPs), and odorant binding proteins (OBPs) expressed by the support cells near the olfactory neurons at the base of the sensilla^24,25‚26^. These proteins are involved in transporting odorants to the ORs at the dendritic interfaces^26,27‚28^. OBP-encoding genes exhibit significant variation in number across insect species, with certain mosquitoes possessing more than 100 genes while some ant species have only 13^28‚29^. Studies indicate that not all *OBPs* have the same function. Some are crucial for the olfactory system, while others have a modulatory impact or no function. Additionally, *OBPs* are found to be associated with various processes, such as odorant release, development, regeneration, and physiological pathways^20,21^. Both OBPs and CSPs are small compact polypeptides composed mainly of α-helical domains and all these proteins possess hydrophobic binding cavity. The structure of OBPs is characterized by three disulfide bridges (interlocked) between conserved cysteine residues, in contrary CSPs have two disulfide bridges between adjacent cysteines^20,29‚21‚30^. Interestingly it was found that some members of the *CSP* family, especially Sensory Appendage Proteins (*SAPs*) are upregulated upon insecticide exposure. A recent study on *A. gambiae* demonstrated that *SAP2* is highly expressed in the legs of pyrethroid (commonly used insecticide of mosquito net) resistant mosquitoes^30‚31^. It was further observed that overexpression of the *SAP2* results in resistance to pyrethroid, and knockdown of gene results in pyrethroid susceptibility, indicating a direct relation between *SAP2* and pyrethroid resistance.

Apart from the adult stages, *Anopheles* mosquitoes also depend on olfaction during the vulnerable larval stage for navigation and survival. This is achieved through the expression of olfactory receptors in the antennae and maxillary palps, although larval olfaction is still very poorly understood^31‚32^. A strong aversive response to harmful compounds such as acetophenone (produced by *Pseudomonas*), DEET (a potent adult mosquito repellent)^32‚33^, and mosquito larval repellent VUAA1^33‚34^ suggests *Anopheles* larvae possess chemosensory receptors. Electrophysiological studies of the larval antennae revealed the population response properties of larval olfactory neurons^34‚35^. The larval antenna is composed of a sensory cone and peg organ which are essential for olfaction and gustation^35‚36^.

Since olfactory genes serve a variety of functions in mosquitoes, identifying and understanding the structure and function of these genes presents a lucrative target for developing effective countermeasures to develop effective vector control strategies. We conducted this study to identify and catalog all olfactory genes of two major Indian malaria vectors and two African malaria vectors. Additionally, we delved into the evolutionary aspects of these genes, examining gene collinearity, domain duplication, and the selection pressure acting on these genes. Moreover, we pinpointed crucial odorant-binding proteins (*OBPs*) specific to different life stages, sexes, and post-plasmodium infection in the mosquito vector. We also examined the expression of *SAP* and *CSP* in both larval and adult stages of *Anopheles stephensi*, and our findings suggest that SAP2 is likely associated with insecticide resistance.

## Results and discussion

### Identification and cataloging of the olfactory genes and synteny analysis

**Fig. 1:**
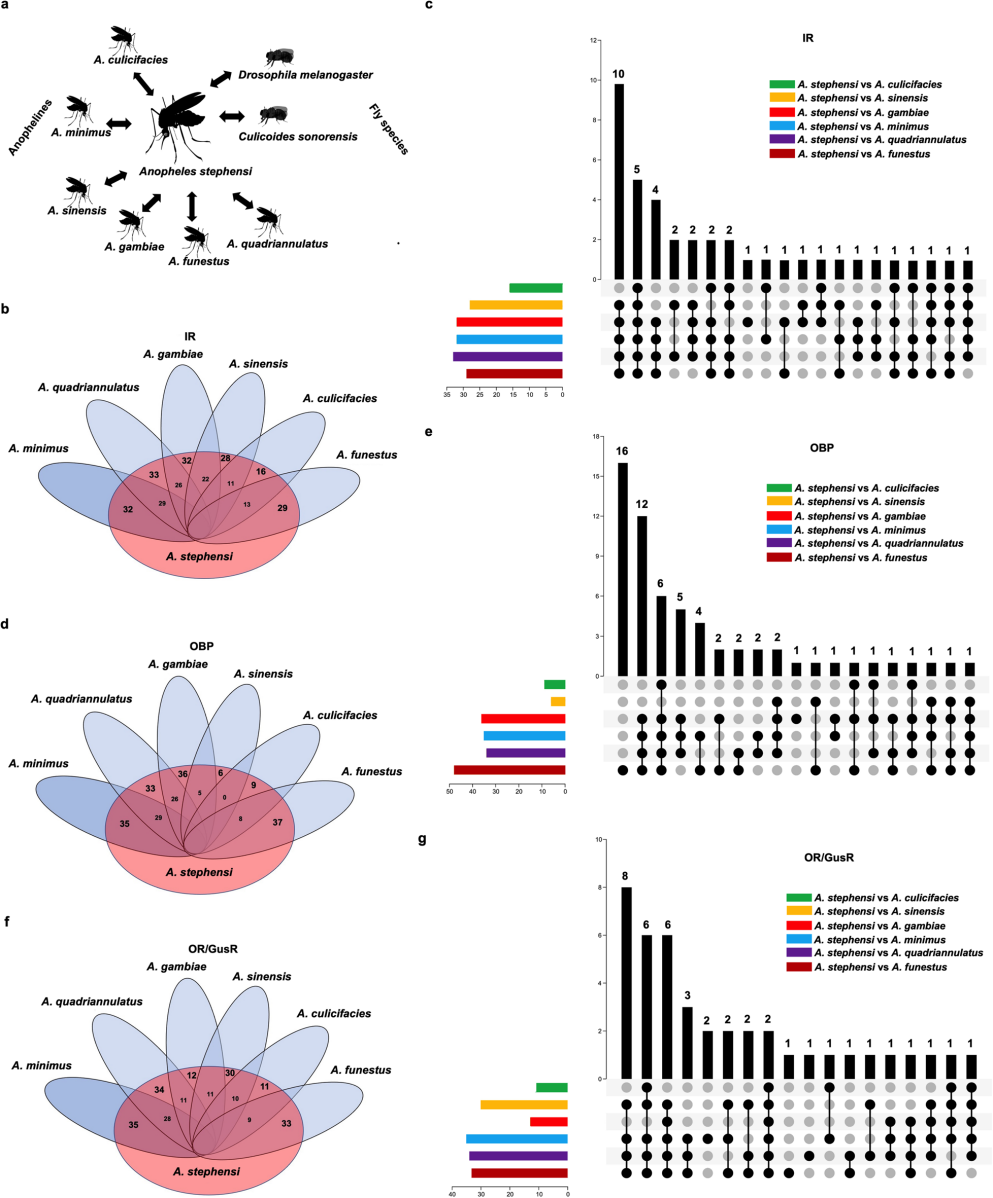
Synergy and Divergence in Olfactory Gene Synteny: A Comparative Analysis of *A. stephensi* and Other *Anopheles* Species, (**a**) Diagram illustrating the synteny analysis conducted between *A. stephensi* and other species. (**b**–**g**) Venn diagrams (left) depicting the results of the synteny analysis. This analysis identified collinear blocks between *IRs*, *OBPs*, and *OR/GusR* in *A. stephensi* and six other Anopheles species. None of the *IRs*, *OBPs*, or *OR/GusR* from *A. stephensi* were homologous to those in any of the fly species. The numbers between the Venn diagram sections indicate the count of syntenic genes shared between the respective species. The bar plots (right) provide a detailed synteny comparison of *IRs*, *OBPs*, and *OR/GusR* between *A. stephensi* and *A. gambiae*, *A. quadriannulatus*, *A. minimus*, *A. sinensis*, *A. funestus*, and *A. culicifacies.*

In this study, we systematically cataloged olfactory genes—namely *OBPs*, *ORs*, *IRs*, and *GusRs*—in two Indian malaria vectors (*A. stephensi* and *A. culicifacies*) and two African malaria vectors (*A. gambiae* and *A. funestus*) (see Supporting Information Figure S1a-b and Table S1, SX1-SX2). Following this, we analyzed the evolutionary conservation of gene order among these olfactory genes through synteny analysis. We compared the genome of *A. stephensi* with those of six other Anopheline species (*A. gambiae*, *A. minimus*, *A. culicifacies*, *A. quadriannulatus*, *A. funestus*, and *A. sinensis*) and included two fly species (*D. melanogaster* and *C. sonorensis*) to assess gene order conservation within mosquitoes and related taxa, as Culicoides species also require host blood for their lifecycle (Fig. 1a).

Our synteny analysis revealed that *OR* genes exhibited higher collinearity between *A. stephensi* and other Anophelines, except for *A. culicifacies* (Fig. 1b and c). In contrast, no collinearity was observed between *A. stephensi* and the fly species for any olfactory genes. For *OBPs*, *A. stephensi* showed the highest collinearity with *A. gambiae* (36 genes), followed by *A. minimus* and *A. quadriannulatus*, with the least collinearity observed with *A. sinensis* (6 genes) (Fig. 1d). None of the *OBP* genes were found in all comparisons (detailed comparisons for *OBPs* are presented in Fig. 1e). For *ORs* and *GusRs*, the highest collinearity was seen between *A. stephensi* and *A. minimus*, followed by *A. quadriannulatus* and *A. funestus*, while the lowest was with *A. culicifacies* (Fig. 1f). Notably, the number of syntenic *ORs* between *A. stephensi* and *A. gambiae* was relatively lower compared to other comparisons (Fig. 1f–g).

### Olfactory gene distribution in Indian Malaria vectors (Invasive)

**Fig. 2:**
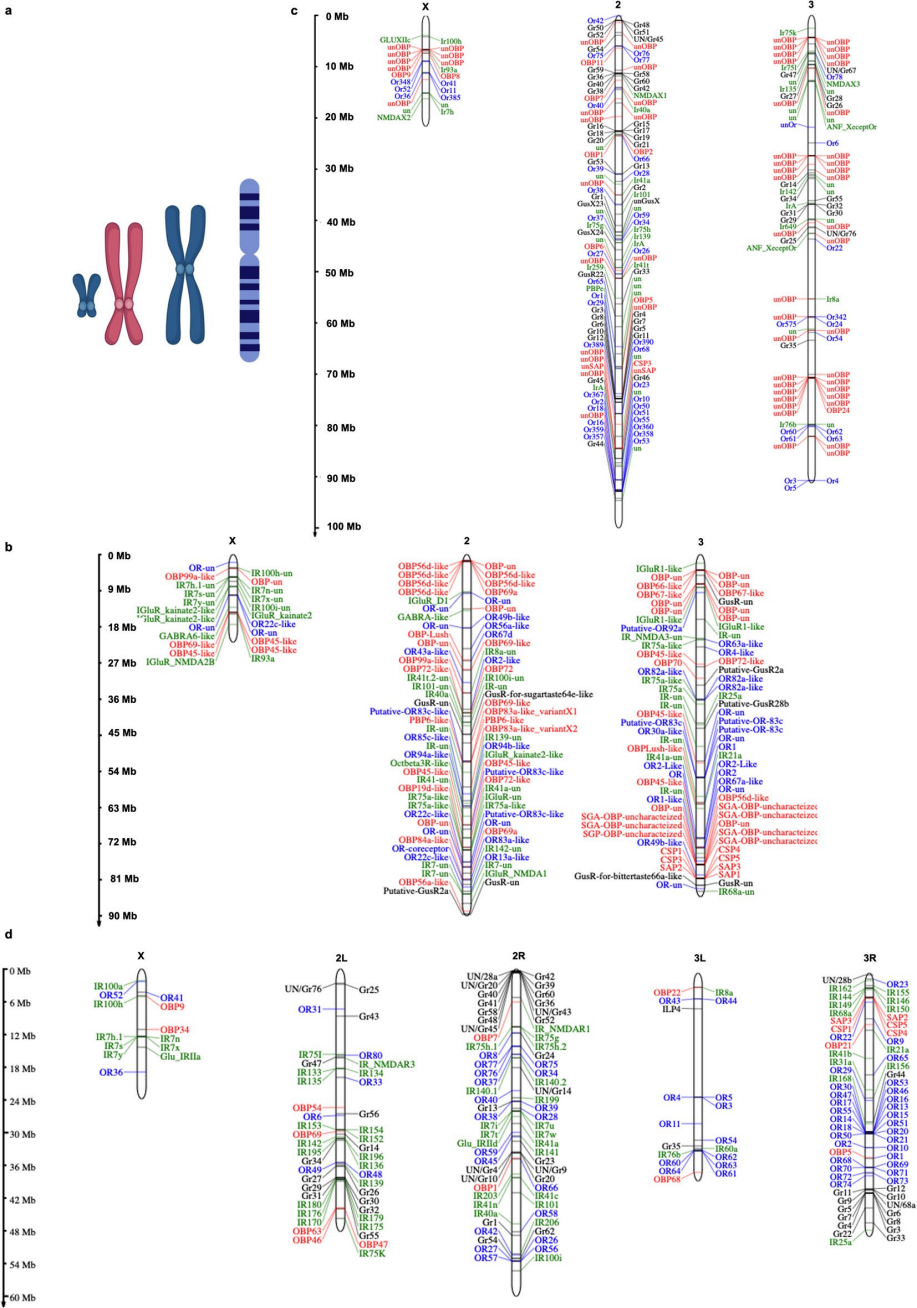
Chromosomal Distribution and Localization of Olfactory Genes in *A. stephensi*, *A. gambiae*, and *A. funestus*, (**a**) Schematic diagram illustrating the different chromosomes present in the mosquito. (**b**) Chromosomal map of *OBPs*, *ORs*, *GusR*, and *IRs* in *A. stephensi*. (**c**) Localization of *OBPs*, *ORs*, *GusRs*, and *IRs* within the *A. gambiae* chromosome. (**d**) Distribution of *OBPs*, *ORs*, *GusRs*, and *IRs* across the chromosomes of *A. funestus*. *OBPs* are depicted in red, *ORs* in blue, *GusR* in black, and *IRs* in green. The scale representing the total length of each chromosome is shown on the left side of the figure.

In our catalogue, we identified 72 odorant-binding proteins (*OBPs*) in the genome of *A. stephensi*. Of these, 4 were chemosensory proteins (*CSPs*), 3 were sensory appendage proteins (*SAPs*), and the remaining were classified as *OBPs* (Figures S1a-b and Table S1). The detailed gene structure of the selected genes (*CSPs*) is given in Figure S1c. Previous studies had reported a total of 44 *OBPs* in *A. stephensi*^36‚37^. For 4 of the 72 *OBPs*, chromosomal location data was not available on VectorBase; these unidentified *OBPs* are listed in Table SX1. Regarding other olfactory receptors, we identified 53 ionotropic receptors (*IRs*), 47 odorant receptors (*ORs*), and 9 gustatory receptors (*GusRs*) in *A. stephensi* (Figures S1b and Table SX1). The chromosomal locations of these genes are detailed in Fig. 2. Chromosome 3 of *A. stephensi* is the predominant location for a significant portion of olfactory-related genes, housing 33 out of 72 *OBPs*, 22 out of 47 *ORs*, 24 out of 53 *IRs*, and 5 out of 9 *GusRs* (Fig. 2). Notably, approximately 24% of the *IR* genes (13 out of 53) are located on chromosome X, a higher proportion compared to other gene types; only around 8% of *ORs* and *OBPs* are found on chromosome X, and no *GusRs* are located there. Chromosome 2 shows the highest clustering of olfactory genes (Figure S1b and Fig. 2). In the *A. culicifacies* genome, we identified 67 *OBPs*, 83 *IRs*, 53 *ORs*, and 57 *GusRs*. However, due to the fragmented nature of the *A. culicifacies* genome, many of these olfactory genes remain uncharacterized, particularly with respect to their chromosomal locations. The genes are listed in Table SX1. Due to the large number of scaffolds (> 20,000), a comprehensive gene location map for *A. culicifacies* could not be constructed.

### Olfactory gene distribution in African malaria vectors (non-invasive)

Our catalogue reveals a higher number of olfactory genes in African malaria vectors compared to Indian vectors (Figure S1a). In *A. gambiae*, we identified a total of 86 *OBPs*, 108 *IRs*, 83 *ORs*, and 57 *GusRs* (Table S1 and SX2). Notably, 60% of the *IRs* in *A. gambiae* are located on Chromosome 2, where they are predominantly organized into gene clusters (Fig. 2). This clustering suggests frequent duplication events followed by functional specialization^37‚38^. In *A. funestus*, we identified 81 *OBPs*, 68 *IRs*, 66 *ORs*, and 57 *GusRs* (Table SX2). Similar to *A. gambiae*, many of these genes are clustered in the genome. Specifically, 65% of the *ORs* are located on Chromosome 2 (Fig. 2 and Table S1).

Our investigation reveals notable differences in olfaction-related gene counts between African and Indian malaria vectors. Specifically, African vectors—*A. gambiae* and *A. funestus*—exhibit an average increase of approximately 19% in *OBPs*, 32% in *ORs*, 27% in *IRs*, and a substantial 72% in *GusRs* compared to Indian malaria vectors—*A. stephensi* and *A. culicifacies* (Figure S1a). This increased gene repertoire in African vectors highlights the potential evolutionary significance of olfaction in these malaria-carrying mosquitoes. Remarkably, these findings are consistent with a previous study^38‚39^, which reported a similar increase in *OR* gene numbers specifically in African vectors. Additionally, the genome of *A. funestus* displayed alignment patterns more similar to Indian Anopheles species than to other African species^38‚39^. This unique genomic alignment suggests that *A. funestus* may have a more complex olfactory behavior, potentially indicating a distinct olfactory mechanism among African Anopheles species.

Given the crucial role of odorant-binding proteins (OBPs) in chemoreception and their potential as targets for disrupting insect chemosensory systems, we concentrated our further analysis specifically on OBPs. We recognized their diverse functions throughout the mosquito life cycle, underscoring their importance in our study.

### Domain analysis of odorant binding proteins (OBPs)

**Fig. 3:**
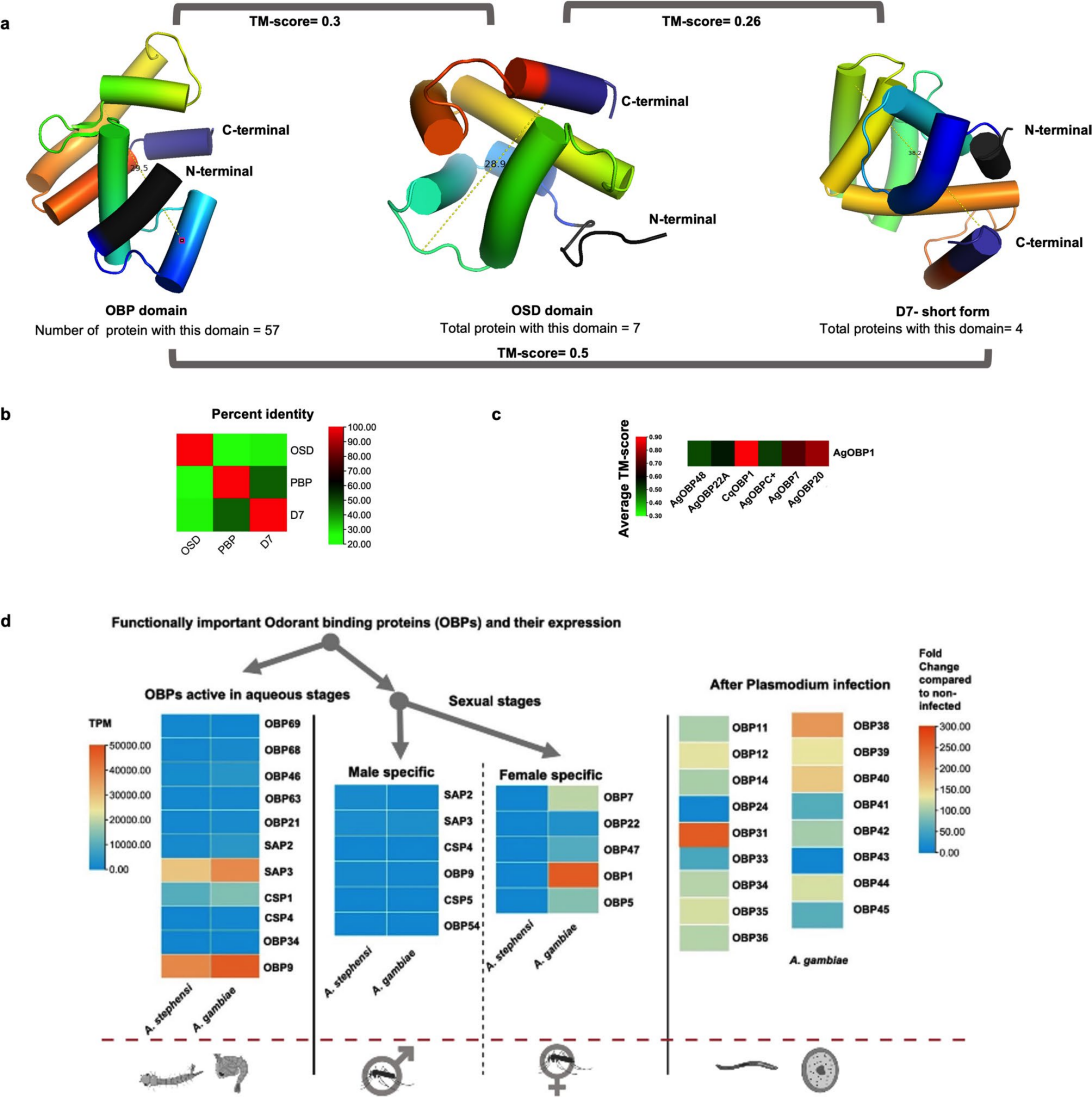
Structural and Expression Analysis of Odorant-Binding Proteins (OBPs), OSD, and D7 domains in Mosquitoes, Structural difference between *OBP* (*PBP-GOBP*), *OSD*, and D7 domains. (**a**) Shows the protein structure of OBPs (PBP_GOBP), OSD, and D7 short-form domains. The N-terminal of the protein is shown in dark grey color, while the C-terminal is shown in dark blue color. The TM score shows structural (fold) similarity between two proteins with “0” meaning they are completely different and “1” meaning similar. (**b**) Heatmap showing the percent identity of the amino acid sequences of PBP_GOBP, OSD, and D7 short-form domain. (**c**) Heatmap showing the structural similarity (TM-score) between different available structures of OBPs. The structures were downloaded from the PDB database. (**d**) Characterizing important odorant-binding proteins (OBPs) based on their expression during aquatic stages, sex-specific patterns, and following *Plasmodium* infection. The expression data was carefully curated from VectorBase, and the relative Transcripts Per Million (TPM) values were represented as a heatmap. The TPM values of corresponding stages were obtained for both *A. stephensi* and *A. gambiae*. To identify the odorant-binding proteins (*OBPs*) expressed after *Plasmodium* infection, the authors obtained fold change transcriptomics data from a previous study^40‚41^. The fold change data were available only for *A. gambiae.*

The catalog of OBPs from our study has two types of protein domains: either Pheromone Binding Proteins/Global Odorant Binding proteins (PBP_GOBP) or Olfactory specific genes D (OSD) (Table SX1 & SX2). The tertiary structure of insect odorant-binding proteins (OBPs) is well-defined, comprising six alpha-helices that are stabilized by three disulfide bonds, creating a binding cavity that is surrounded by hydrophobic residues. Even though they are not similar in sequence and structure, the OSD protein domains share a few features common to odorant-binding proteins. (Fig. 3a and b). The OSD domain is present in Chemosensory proteins (CSPs) and sensory appendages proteins (SAPs) in *Anopheles* mosquitoes (Table SX1 and SX2).

OBPs are a highly diverse class of proteins, and the biggest variation of this OBP domain can be found in the D7 class of protein, which is found abundantly in the salivary gland of blood-feeding Nematocera. The D7 protein domains like OBPs are made up of alpha-helices, usually consisting of 7–8, which is slightly more than the typical 6 found in OBPs. These alpha-helices combine to form a hydrophobic pocket, which allows them to bind hydrophobic molecules effectively^39‚40^. The D7 domains have a wider cavity (~ 10 nm more) than OBP suggesting a potential binding with a wider variety of ligands. The sequence similarity was very less between these three domains (OBP, D7, and OSD) as shown in Fig. 3b. To further assess their structural similarity, we calculated the Template Modelling Score (TM-score), which was < 0.6 for all comparisons, indicating minimal structural similarity among them (Fig. 3a). Furthermore, we have also calculated structural variation within the OBP class of proteins based on their existing crystal structure. Our analysis clearly revealed that AgOBP1 exhibited significant structural similarity to CuOBP1, AgOBP7, and AgOBP20, respectively. Conversely, AgOBP48, AgOBP22A, and AgOBPC + displayed a distinct structure from AgOBP1, as evidenced by their TM score (Fig. 3c).

Domain structural variation suggests that OBPs can accommodate a range of ligands in their binding pockets, potentially triggering various biological events beyond olfaction. This variability may influence multiple aspects of mosquito biology and their interactions with parasites. Further research into the specific mechanisms and pathways involved will deepen our understanding of how *OBPs* impact mosquito survival and the dynamics of parasite transmission.

### Functional characterization of important *OBPs*

To explore the roles of *OBPs* beyond olfaction, we analyzed publicly available transcriptome data for *A. stephensi* and *A. gambiae* from VectorBase and previous studies. Our goal was to identify key *OBPs* involved in mosquito development and to categorize them based on their expression patterns across both aquatic and terrestrial stages of the mosquito life cycle. We also investigated whether *OBP* expression changes following infection with *Plasmodium falciparum*, the malaria-causing parasite (see Fig. 3d and Table S2). Additionally, we examined the potential involvement of *OBPs* in insecticide resistance.

### *OBPs* expression during aquatic life stages

A total of 11 *OBPs*/*CSPs* were found to be showing a higher expression in aquatic life stages as compared to terrestrial stages. These include 4 Classic OBPs, 2 Atypical, 3 minus C, 1 Plus C, and 1 Dimer having a PBP-GOBP (IPR036728) or OSD (IPR005055) domain (Table S2). In general, OBPs are classified based on the presence of conserved cysteine residues resulting in different classes, such as classic, atypical, plus-C, minus-C OBPs, and dimer OBPs^36‚37^. The classic OBPs contain six conserved cysteines, with cysteine 2 (C2) and cysteine 3 (C3) being conserved and three amino acid residues apart. Similarly, cysteine 5 (C5) and cysteine 6 (C6) are conserved, but eight amino acid residues separate them. However, the number of amino acids between C1-C2, C3-C4, and C4-C5 can vary. While classic and atypical OBPs contain the same number of conserved cysteines, the number of amino acids between them differs. Plus-C OBPs have two additional cysteines, 4a and 6a, along with a conserved proline immediately after C6. The dimer OBPs have a two-cysteine signature^28,36‚29‚37^. In the aquatic stage, the highest expression was found in *OBP9* (TPM = 4021 in *A. stephensi* and TPM = 4089 in *A. gambiae*) and *SAP3* (TPM = 2970 in *A. stephensi* and TPM = 3975 in *A. gambiae*) followed by *CSP1*, *SAP2*, *OBP46* and rest (total 11) (Fig. 3d & Table S3).

### *OBPs* expression pattern in male and female mosquitoes

A total of 6 male-specific *OBPs* and 7 female-specific *OBPs* were identified by analyzing the transcriptomics data on the VectorBase having a comparatively higher expression in the respective stages (Table S3). Among the male-specific *OBPs*, *SAP3* displayed the highest expression level relative to other *OBPs*. Among the female-specific *OBPs*, *OBP1* had the highest expression level, followed by *OBP7*, *OBP5*, and *OBP47*, respectively (Fig. 3d). The values of TPM for each of these genes in *A. stephensi* and *A. gambiae* are given in Table S3. There was no evidence of domain duplication in any of the sex-specific *OBPs* we examined. Moreover, the Ka/Ks ratio in these genes was less than 1, indicating the presence of purifying selection. However, the positive selection was found in 2 *OBPs i.e., SAP2* (Male-specific) and *OBP22* (Female specific) on the amino acid level. The *SAP2* has 2 positively selected sites and in *OBP22*, only 1 positively selected site was found (Table S2).

### *OBPs* expression after *Plasmodium* infection

From a previous study^40‚41^, we identified 17 *OBPs* that exhibited a significant increase in the expression levels in mosquitoes infected with *P. falciparum*, with the pattern observed after 7 days from the infections. Among these, *OBP31* exhibited the highest expression level with a fold change of 262.67, while *OBP43* had the lowest expression with a fold change of 6.62. Additionally, *OBP34*, which has domain duplication in *A. stephensi* and *A. minimus* (as previously explained), showed a 110-fold higher expression on the 7th day compared to uninfected control mosquitoes (Fig. 3d). For each of these *OBPs*, the fold change values are given in Table S3. Out of the 17 *OBPs*, 8 showed the domain duplication event while 9 had a single *PBP-GOBP* domain in the protein. After the infection, the expression of nine odorant-binding proteins (*OBPs*) namely *CSP5*, *CSP4*, *OBP3*, *OBP2*, *OBP26*, *OBP29*, *OBP46*, *OBP21*, and *OBP69*, was found to be downregulated after seven days of infection. However, the decrease in expression was only observed to be around one-fold, ranging from − 1.01 to − 1.42-fold^40‚41^.

### *OBPs* expressions and insecticide resistance

Given the crucial role of *OBPs* in mosquito olfaction and development, as well as their emerging link to insecticide resistance, we conducted a targeted investigation into the expression patterns of specific *OBPs* during the aquatic stages of *A. stephensi*. Our study focused on *OBPs*, including both *CSPs* and *SAPs*, which have recently been highlighted for their involvement in olfactory processes and insecticide resistance, as noted in a recent study^30‚31^. We specifically examined the aquatic stages of an insecticide-resistant strain to identify potential variations in *OBP* expression. The chromosomal map and gene structure of the selected *OBPs* are shown in Figure S1c.

To check if the *CSPs* and *SAPs* are expressed in larval and pupal stages, we extracted total RNA (3 biological cohorts) from deltamethrin resistant as well as susceptible strains of *A. stephensi*. In the susceptible strain of *A. stephensi*, male pupae exhibited notably higher expression levels of CSPs compared to female pupae, as depicted in Figure S2. Conversely, in the resistant strain, a significantly higher expression of *CSPs* was observed in L2 larvae when compared to L1 larvae Figure S2. Although expression of *CSP5* was significantly higher in all larval stages, its expression in male and female pupae have approximately 40-fold increase suggesting that *CSP5* plays an important role in olfaction as pupae are about to emerge as adults and their olfactory system is activated to overcome the need for finding food source as well as a mate after emergence. A recent study in the cotton aphids, *Aphis gossypii*, showed a link between agos*CSP5* higher expression to the insecticide resistance^41‚42^. We saw a similar pattern in the *A. stephensi* deltamethrin-resistant strain where expression of *CSP5* was higher in all aquatic stages, especially in pupae as compared to the deltamethrin-susceptible strain. *CSP3* was not included in the experiment because it was not detected in either the larval or pupal stages of *A. stephensi*.

**Fig. 4:**
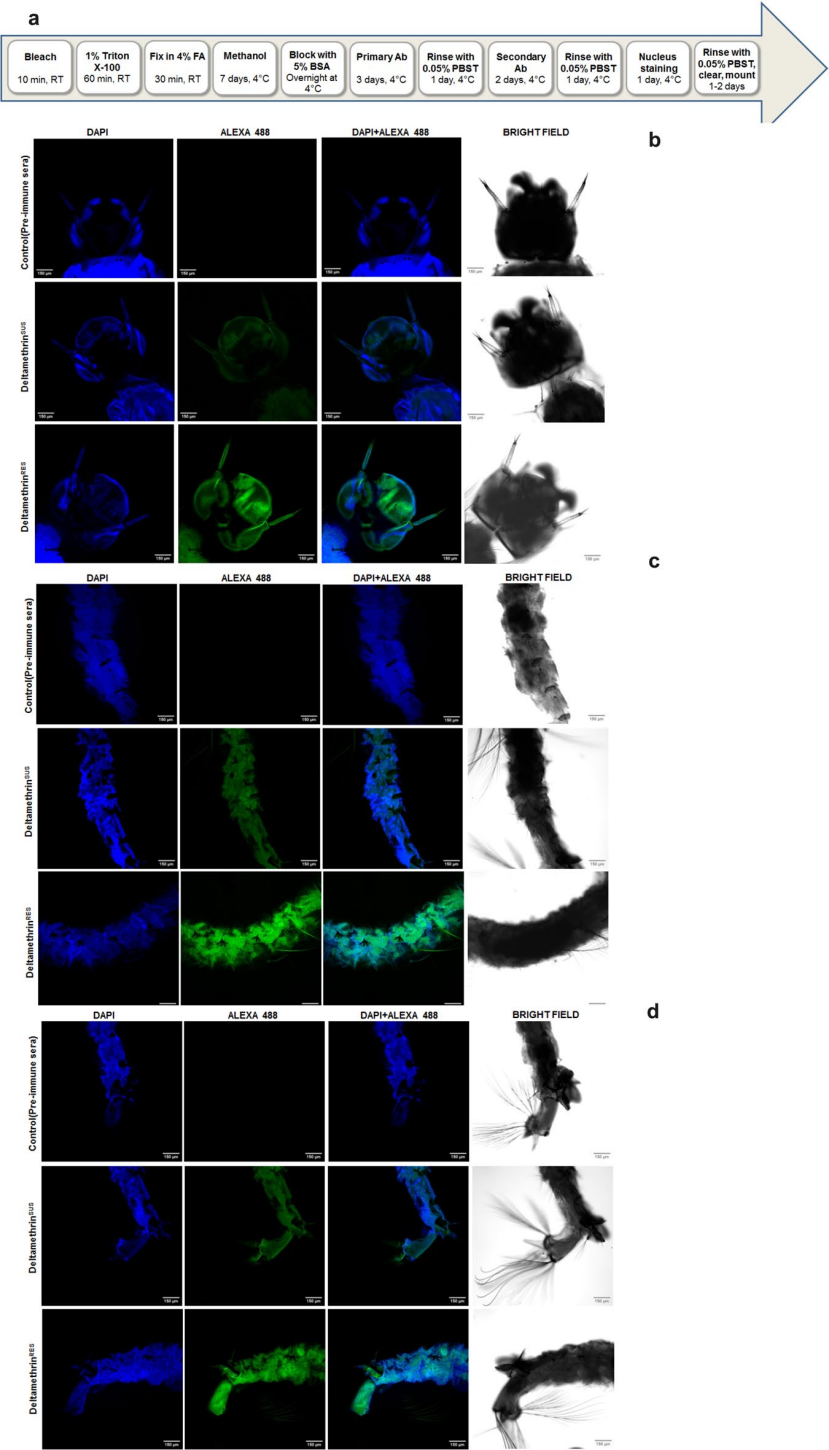
Immunofluorescence Analysis of SAP2 Expression in A. stephensi L3 Larvae from Deltamethrin-Susceptible and Resistant Strains, (**a**) Schematic presentation of the methodology for preparing A. stephensi L3 larvae for immunofluorescence: Abbreviations used in the schematic: FA: Formaldehyde, Methanol; BSA: Bovine Serum Albumin (5%); Primary Ab: Pre-immune mouse serum or Anti-SAP2 antibody (1:1000 dilution); Secondary Ab: Goat anti-mouse IgG conjugated with Alexa Fluor 488 (1:2500 dilution); PBST: Phosphate-buffered saline with Tween-20 (0.05%); Nucleus staining: DAPI (1:300 dilution). Imaging of third-instar larvae (L3) of *A. stephensi* from both deltamethrin-susceptible and resistant strains, stained with DAPI (blue) and Alexa Fluor 488 (green; SAP2) at 10 × magnification. Differential Interference Contrast (DIC) and stained images are shown for the following regions of L3 stage larvae: (**b**) antennae and head region, c) mid-region, and d) tail region of both deltamethrin-susceptible (Deltamethrin^SUS^) and deltamethrin-resistant (Deltamethrin^RES^) strains. Control samples were incubated with mouse pre-bleed serum instead of Anti-SAP2 antibody (1:1000 dilution).

Moving on to *SAPs*, we observed a remarkable 25-fold increase in the expression of *SAP2* and a 15-fold increase in the expression of *SAP3* in the resistant strain compared to the susceptible strain. Notably, this heightened expression was predominantly observed in L2-stage larvae, after which the expression decreased in subsequent larval stages (Figure S2). The expression of *SAP2* was also found higher in the adult stages (male and female) of deltamethrin resistant strain. Our findings align with previous reports on *A. gambiae*, where *SAP2* was found to be elevated in pyrethroid-resistant strains^30‚31^. In the case of *SAP1*, the expression remained relatively similar between the two strains, except for male pupae, where higher expression of *SAP1* was observed in the susceptible strain compared to the resistant strain (Fig. 4a–d). The *CSPs* and *SAPs* were also found to be expressed in the aquatic as well as adult stages of *A. culicifacies* (deltamethrin susceptible strain), especially in the case of pupae, where expression in males was significantly higher than in females. Our findings provide further validation for the potential involvement of *CSPs* and *SAPs* in insecticide resistance, extending beyond their previously established roles in adult mosquitoes to encompass the aquatic stages as well.

### Immunofluorescence shows higher SAP2 expression in resistant larvae

Quantitative transcription studies revealed heightened expression of *SAP2* and *SAP3* in all the aquatic life stages of deltamethrin resistant *A. stephensi* in comparison to the aquatic stages of the susceptible strain. To validate the transcription data, immunofluorescence was performed against L3 stage of both susceptible and resistant (deltamethrin) larvae of *A. stephensi*. For immunofluorescence polyclonal antibodies were raised in mice against recombinant SAP2 protein. The effective titer of SAP2 (for immunofluorescence) antibody was determined through immunoblots assay. Native SAP2 protein expression was monitored by intensity of green colored fluorescence appeared in the images (as the secondary antibody was labeled to Alexa Fluor 488 fluorophore). The immunofluorescence comparison of the L3 larvae (both deltamethrin susceptible and resistant strain) clearly shows significant higher expression of SAP2 in the resistant variants compared to susceptible one, confocal imaging data further supports our earlier expression data based on q-RT-PCR (Fig. 4a–d and Figure S2). In resistant L3 larvae high SAP2 expression was observed throughout different body parts including antennae and head region, mid-gut area as well as tail-region (Fig. 4a–d). The blue fluorescence appears due to DAPI staining indicative of optimum permeabilization of the dye in the larvae. Both immunofluorescence and transcriptome-based expression data clearly suggests a direct correlation between *SAP2* expression and insecticide resistance in *A. stephensi*. Using the same SAP2 antibody, we also examined the expression of SAP2 in the legs of adult insecticide-resistant mosquitoes and found that SAP2 protein was highly localized and overexpressed in resistant mosquito legs compared to those of susceptible mosquitoes (Figure S3). This observation is consistent with findings reported by Ingham et al.^30‚31^ in adult *Anopheles gambiae*, and further supports the hypothesis that these genes may begin to be primed prior to the adult stage, potentially contributing to the development of resistance in adult mosquitoes.

### Phylogenetic and mutation analysis of OBPs related to insecticide resistance

To further explore the role of CSPs and SAPs in insecticide resistance, we examined the evolutionary relationships of these genes by constructing a maximum likelihood (ML) phylogenetic tree. We used amino acid sequences from seven *Anopheles* species (*A. stephensi*, *A. gambiae*, *A. quadriannulatus*, *A. minimus*, *A. sinensis*, *A. funestus*) and two *Culicine* species (*Ae. aegypti* and *C. quinquefasciatus*), with OBP 22 included as a control. The phylogenetic tree, shown in Figure S4a, revealed a distinct clade for *OBP 22* and *CSPs*. However, SAP2 and SAP3 appeared intermixed due to their high sequence similarity (Figure S4a). Additionally, we analyzed mutations in the selected genes using sequence data from the Agam1000 database, which includes field-collected samples of *A. gambiae*. We observed a notably high mutation rate in CSP3 among these field-collected samples (Figure S4b), suggesting that CSP3 may experience significant genetic variation in natural populations of *A. gambiae*.

### Domain duplication events in *OBP*

Our analysis revealed considerable structural and functional diversification among *OBPs*. In *Drosophila*, gene duplication is a key evolutionary mechanism driving both structural diversity and neofunctionalization of *OBPs*^42‚43^. Until now, the role of gene duplication in the diversification of *OBPs* in mosquitoes was not well understood. During our cataloging process, we identified several instances of gene duplication within *OBPs*. Given that domain duplication is a significant event in insect biology, we investigated this phenomenon further. Our analysis of domain duplication events among OBP paralogs in *A. stephensi*, *A. culicifacies*, *A. gambiae*, and *A. funestus* highlights the dynamic and evolving nature of these OBPs in mosquitoes (Figure S4c). The extent of domain duplication varied across these species, ranging from 7 to 19 duplicated domains. *A. gambiae* exhibited the highest number with 19 duplicated domains, while *A. funestus* had the lowest with 7. In the two Indian malaria vectors, *A. stephensi* and *A. culicifacies*, we identified 9 and 11 OBPs with duplicated domains, respectively. Figure S3d shows the OBPs with duplicated domains, marked in red before their respective gene IDs.

Among the various OBPs, we observed a notable duplication event in OBP34 (which also exhibits high transcriptomic expression during the aquatic stage), where additional PBP_GOBP domains were present in *A stephensi* (Fig. S5). To explore the evolutionary implications of this finding, we examined the orthologs of OBP34 in seven other Anopheles species. Our investigation revealed that duplication also occurred in *A. minimus*, suggesting that this event may have arisen independently in multiple species rather than being unique to *A. stephensi* (Figure S5). The domain duplication observed in both *A. stephensi* and *A. minimus* likely indicates functional diversification^43‚44^. Domain 1 of OBP34, named based on its position from the N-terminal, appears to be the ancestral domain, as supported by the Maximum Likelihood phylogenetic tree constructed from domain sequences of other Anopheles species. Domains 2 and 3, which are identical to domain 1, suggest duplication without significant diversification (Figure S5). The Ka/Ks ratio, comparing the rates of synonymous and nonsynonymous substitutions, was less than 1 for all 11 OBPs (Table S1). Notably, we did not detect any significantly positively selected amino acids in the OBP34 (Figure S5).

Interestingly, domain duplication in some OBPs results in highly diversified domains. To investigate this phenomenon, we conducted a structural comparison of the original and duplicated domains using the template modeling (TM) score. This analysis revealed that similarity between original and duplicated domains varied among OBPs. The highest TM score of 0.8 was observed in ASTEI20_044028 (uncharacterized OBP), indicating a strong structural resemblance between domain 1 and domain 2. In contrast, the lowest TM score of 0.4 was recorded in ASTEI20_045100 (uncharacterized OBP), suggesting a lower similarity between its domains. Overall, TM scores for both original and duplicated domains were consistently below 0.6 (Figure S6a), highlighting distinct structural differences within these domains. These results suggest that domain 2 tends to be more diversified compared to domain 1 (Figure S7).

To further distinguish domain duplication event between domain 1 and domain 2, we analyzed a phylogenetic tree with OBP1 and OBP10 of *A. stephensi* as references, as they each possess a single PBP-GOBP domain. Maximum likelihood (ML) tree analysis strongly supports domain 1 as the original domain and domain 2 as the duplicated one (Figure S6a). Notably, domain 2 in *A. stephensi* showed significantly less similarity to domain 1, as indicated by both the ML phylogenetic tree and multiple sequence alignments of the domain sequences (Figure S6b).

## Conclusion

This study for the first time provides a comprehensive detail of odorant binding proteins and olfactory receptors involved in the sense of smell in major Indian and African malaria vectors. Apart from detail cataloging of olfactory genes, we identified *OBPs* important for aquatic life stages of mosquitoes and *Plasmodium* infection. By investigating domain duplication and positive selection events, we gained valuable insights into the potential future evolution and functions of these genes. By providing a more complete picture of the genes responsible for olfaction in Anopheline vectors, this study will help to advance our present molecular level understanding of the mechanisms involved in host-seeking and blood-feeding behaviors. The ecological success of mosquito species depends on their ability to face the challenges of a new environment. Evolutionary forces, *e.g.,* migration, can shape the adaptive process, and among the most important adaptation mechanisms are host-seeking and feeding behavior. Developing an olfactory catalog for invasive and noninvasive vectors will shed light on the vectorial capacity of these species.

Furthermore, our findings offer additional evidence supporting the involvement of chemosensory proteins (*CSPs*) and sensory appendage proteins (*SAPs*) in insecticide resistance, expanding their known roles beyond adult mosquitoes to include the aquatic stages. Particularly noteworthy is the expression of *SAP2* in the insecticidal-resistant strain in *A. stephensi* (Table S4). The presence of *SAP2*-associated insecticide resistance in Indian Anopheles mosquitoes’ parallels observations in African counterparts, indicating similarities in resistance mechanisms across continents. It is intriguing to note that *SAP2* shows prominent expression not only during the adult stage but also throughout the larval phase. This finding indicates the potential utility of *SAP2* as a marker for insecticide resistance in both aquatic and terrestrial stages of *Anopheles* mosquitoes in future studies. Such a marker can greatly enhance our ability to monitor and manage insecticide resistance in malaria vector populations, enabling more effective control strategies, which in turn can inform the development of more effective vector control methods. Overall, this study has significant potential to contribute to the understanding and management of mosquito-borne diseases. A ‘super mosquito’ from Asia is invading African cities — and it could ignite a dangerous new wave of malaria in the continent’s rapidly growing urban centers. The establishment of *A. stephensi* in Africa poses a potential threat to malaria control and elimination, which further has a global importance and impact.

## Methodology

### Mosquito rearing

Cyclic colonies of *A. stephensi* (Deltamethrin resistant strain) and *A. culicifacies* (sibling species A) were maintained at 28 °C and 80% relative humidity (RH) with a photoperiod of 16 h light and 8 h dark in central insectary facility at ICMR-National Institute of Malaria Research, New Delhi, India^44‚45^. *A. stephensi* deltamethrin susceptible strain was acquired from insect rearing facility of BITS-Pilani, Rajasthan, India and was reared in the CIF, ICMR-NIMR same as the resistant strain. All protocols for rearing and maintenance of the mosquito culture were approved by the ethical committee of the institute.

### Adult susceptibility assay

Susceptibility tests of adult Anopheles mosquitoes were performed according to standard protocols (WHO, 2016). Laboratory-adopted female *A. stephensi* mosquitoes were tested against deltamethrin 0.05%. Insecticide-impregnated papers were obtained from the Vector Control Research Unit (VCRU), Universiti Sains Malaysia, Malaysia (http://www.usm.my). For this assay one-to-three-day-old female *A. stephensi* mosquitoes were used, mosquitoes were exposed to commercially manufactured insecticide-impregnated special papers for 1 h in 3 replicates (15–30 mosquitoes/replicate) in presence of one pyrethroid control (PYC). Exposed mosquitoes were directly transferred to special holding tubes (that carries a glucose-soaked cotton pad) for 24 h. The tubes were maintained at insectary in highly humid (70–80%) condition at 27 ± 2 °C. After 24 h of incubation, percent mortality was calculated. Abbott’s formula (Abbott, 1925) was used to calculate correct mortality, WHO scoring guideline was strictly follows for calculating resistance mosquito population (WHO, 2016). RNA was extracted from larvae of the resistant strains and used for quantitative real-time PCR (qRT-PCR) analysis.

### Identification of olfaction-related proteins

To identify all putative the OBPs, we downloaded the protein fasta file of *A. gambiae, A. sinensis, and Aedes aegypti* from the Vectorbase database(Release number 63) and did several rounds of exhaustive BLASTP search in the genome of the *A. stephensi* (GCF_013141755.1). The presence of the functional PBP/GOBP domain was further confirmed using the Pfam web server (http://pfam.xfam.org/). Further, redundant sequences having 100% sequence identity were removed from the list. A similar tactic was used to search for the Odorant receptors (*ORs*), Ionotropic receptors (IRs), and Gustatory receptors (GusRs) in the *A. stephensi* genome. The gene catalog of *A. culicifacies* (GCA_000473375.1), *A. gambiae* (GCA_000005575.2), and *A. funestus* (GCA_003951495.1) genes was done using the same above-mentioned method. The chromosomal location map of olfaction-related genes was visualized using the phenogram tool (http:// visualization.ritchielab.org/phenograms/plot)^45‚46^. The information about their location, gene length, and the exon number was extracted from the vectorbase (https://vectorbase.org). The map detailing genes of *A. stephensi* and *A. culicifacies* was made using TBTools^46‚47^.

### Synteny analysis of olfaction genes

Synteny was carried out using TBTools^46‚47^ using default settings (e value = 1*e^−10^; No of blast hits = 5). TBTools uses MCScanX to find the syntenic regions between the chromosomes of two organisms^47‚48^. MCScanX is an algorithm that is used to scan the multiple genomes and identify putative homologous chromosomal regions by aligning those using genes as anchors. For this study, we compared *A. stephensi* genome against the genome of two fly species (*D. melanogaster* and *C. sonorensis*), two *Culicine* species of mosquito (*Ae. aegypti* and *C. quinquefasicatus*), and five *Anophelines* (*A. culicifacies*, *A. gambiae*, *A. sinensis*, *A. quadriannulatus*, and *A. minimus* to locate the collinear blocks between their genomes. The upset plot of the synteny analysis was generated using TBTools^46‚47^.

### Phylogenetic and mutation selection analysis

The maximum likelihood tree for paralogs and orthologs was constructed using MEGA11^48‚49^. The Jones-Taylor-Thornton (JTT) model with 1000 bootstrap replicates was employed for phylogenetic tree. For the final modification of the tree, we used the Interactive Tree of Life (http://itol.embl.de/). To determine the selection pressure in *A. stephensi* and *A. culicifacies* paralogs, an ML tree for the paralogs of IRs, GusR, ORs, and OBPs in both species was constructed by MEGA11^48‚49^.

The nonsynonymous to synonymous substitution ratios (Ka/Ks) for the clustered functionally important OBPs was validated using the PAML 4^49‚50^. The orthologs gene cluster were downloaded from VectorBase in 7 *Anopheline* and 2 *Culicine* species. The Ka/Ks ratio was used to check the selection pressure on OBPsand Ka/Ks ratio > 1, < 1, or = 1 indicated positive, negative, or neutral evolution, respectively. Additionally, the site-specific positive selection and purifying selection were assessed by using the SELECTON server^50‚51^. For the domain information and its duplication in the clustered OBPs, NCBI`s CDD database^51,52‚53^ and InterPro database were used^53‚54^.

### Bioinformatics analysis of the *P.falciparum* infection in the vector

To identify the odorant-binding proteins (OBPs) expressed after Plasmodium infection, the authors obtained fold change transcriptomics data from Emami et al. from her previously reported study^40‚41^. The sequencing Fastq files were evaluated for overall quality using FastQC. Adapter sequences and low quality were removed using Trimmomatic (version 0.36; parameters: ILLUMINACLIP:TruSeq3-PE.fa:2:30:10 SLIDINGWINDOW: 4:15 MINLEN:70). Reads retained after quality check were aligned to the mosquito reference genome using HISAT2 (version 2.1.0, using default parameters). Reference genome sequence and annotation were downloaded from Vectorbase.org (AgamP4, version12). Gene read counts were generated using HTSeq-count (version 0.9.1; parameters: -s no -t exon -i gene_id -r pos -m intersection-nonempty) and custom bash scripts (available on demand). Differential gene expression analysis was performed in R using DESeq2 package with default parameters. Visualisations were performed using various graphical packages in R.

### Sample collection and RNA extraction

To check the presence of *CSPs* and *SAPs* in *A. stephensi* (deltamethrin susceptible and resistant strain) and *A. culicifacies* (susceptible) aquatic stages, we collected L1, L2, L3, and L4 stage larvae (~ 100 each) as well as male and female pupae (~ 20 each). RNA was extracted from three separate biological cohorts by placing them separately into 1.7 ml tubes and extracting them with TRIzol™ reagent (Invitrogen). The resulting RNA pellet was resuspended in 30 μl of RNase/DNase free water and incubated at 55–60 °C for 10 min. RNA was treated with DNase I from the Direct-zol™ RNA Miniprep Kit (Zymo Research) at RT for 30 min. Columns from this kit were pre-wet with RNA Wash Buffer, briefly centrifuged, then the DNase-treated RNA was added directly to the columns. Manufacturer protocols were followed for subsequent RNA recovery. Total RNA quantity was measured with a Nanodrop spectrophotometer. Purity was determined by 260/280 and 260/230 ratios, with acceptable values in the ~ 1.8 and 2.0–2.2 range, respectively (all samples collected met purity standards). RNA samples were immediately stored at − 80 °C until further use.

### Expression of *CSPs* and *SAPs* in larvae and pupae

The expression of *CSP1*, *CSP4*, *CSP5*, *SAP1*, *SAP2* and *SAP3*, was characterized using quantitative RT-PCR (qRT-PCR). GoScript™ Reverse Transcriptase cDNA synthesis kit (Promega, A5003) was used to convert 1ug of total RNA to cDNA following manufacturer protocol. Briefly, cDNA from different life stages was diluted 5 × with deionized H_2_O before using it as a template in qRT-PCR experiments. One microliter cDNA was used in each 10 μl qRT-PCR reaction. Actin was selected as a control (housekeeping gene) because of its consistent expression in all life stages. Sequences of specific primers used for *CSPs*, *SAPs* and other housekeeping gene are listed in Table S5. Same set of primers were used for PCR amplification in both *A. stephensi* and *A. culicifacies*. Each sample was run in duplicate wells of 96-well plate. qRT-PCR was performed on CFX touch Real-Time PCR Detection system using SYBR green master mix (Takara, Catalogue number RR82WR). All reactions were performed with an initial 5 min at 95 °C, next 40 cycles of 10 s at 95 °C, 15 s at 60 °C, and 15 s at 72 °C, and a melt curve was analyzed at 70–95 °C. Relative expression was calculated using 2^−ΔΔ*Ct*^ method. All data were analyzed using GraphPad Prism 7 software (La Jolla, CA, USA). Relative expression values in different developmental stages were compared with one-way ANOVA, followed by Dunnett’s multiple comparison test. For clustering functionally important *OBPs*, VectorBase^54, 55^ was used to mine the transcriptome data from available studies on *A. stephensi* and *A. gambiae*. The heat plot was generated by using TBTools^46‚47^.

### Preparation of L3 stage larvae samples for immunofluorescence

The L3 stage larvae of deltamethrin susceptible and resistant strain of *A. stephensi* were collected on the fifth day after egg hatching in the mosquito insectary at the ICMR-National Institute of Malaria Research, Dwarka, New Delhi, India. The collected L3 stage larvae were processed as per the procedure mentioned for *Dorsophila melanogaster* larvae immunofluorescence^12‚55^. Briefly, the steps involved in *A. stephensi* L3 stage larvae sample preparation for immunofluorescence are depicted in the form of a schematic representation (Fig. 4a). The prepared larval samples were observed for immunofluorescence using Nikon confocal microscope [Model: Nikon Ti2E, A1R MP (multiphoton ready)].

### Preparation of adult female legs samples for immunofluorescence

The adult females’ mosquitoes of deltamethrin susceptible and resistant strain of *A. stephensi* were collected on the sixth day after emergence in the mosquito insectary at the ICMR-National Institute of Malaria Research, Dwarka, New Delhi, India. The legs of the collected female mosquitoes were dissected and processed similarly as *A. stephensi* larvae for immunofluorescence study (Fig. 4a)^12‚55^. The prepared larval samples were observed using Nikon confocal microscope [Model: Nikon Ti2E, A1R MP (multiphoton ready)].

## Supplementary Information

Below is the link to the electronic supplementary material.

Supplementary Material 1

Supplementary Material 2

Supplementary Material 3

## Data Availability

Detail sequence analysis, synteny data, q-PCR raw reads, and other materials are available from the corresponding author upon reasonable request.
