# Supplementary material for: Olfactory gene dynamics in invasive Indian and non-invasive African malaria vectors at the crossroads of development, infection and resistance
Source: Sci Rep. 2025 Oct 28;15:37696. doi: 10.1038/s41598-025-21404-9 (PMC12568962; doi:10.1038/s41598-025-21404-9)
Supplement: Supplementary file 3 — Supplementary Material 3 [file 41598_2025_21404_MOESM3_ESM.pdf]

Olfactory Gene Dynamics in Invasive Indian and Non-Invasive African Malaria Vectors at the Crossroads of Development, Infection, and Resistance.

Arvind Sharma[1, 2#], Bhuvan Dixit[1#], Bharti Goyal[1,3] Renuka Harit[1,3], Jatin kumar[1,3], Shibani Biswas[1,3], Ritu Goswami[1,3], Kailash C Pandey[1,3], S. Noushin Emami[4,5,6,7*], Soumyananda Chakraborti[1, 8*]

[1]ICMR-National Institute of Malaria Research, Dwarka, New Delhi, India
[2]Department of Biochemistry and Molecular Biology, University of Nevada, Reno, USA
[3]Academy of Scientific and Innovative Research (AcSIR), UP, India
[4]Natural Research Institute, University of Greenwich, Greenwich, UK
[5]Department of Vector Biology, Liverpool School of Tropical Medicine, Liverpool, L3 5QA, UK
[6]Molecular Attraction AB, Elektravägen 10, Hägersten, Stockholm, 126 30, Sweden
[7]Department of Microbiology, Tumor and Cell Biology, Karolinska Institute, Sweden
[8]Department of Biological Sciences, BITS-Pilani(Hyd), Hyderabad, India

[#]Authors contributed equally
*Author for correspondence:
SC: soumyananda.chakraborti@hyderabad.bits-pilani.ac.in
SNE: S. Noushin Emami: noushin.emami@lstmed.ac.uk

Supporting Figures

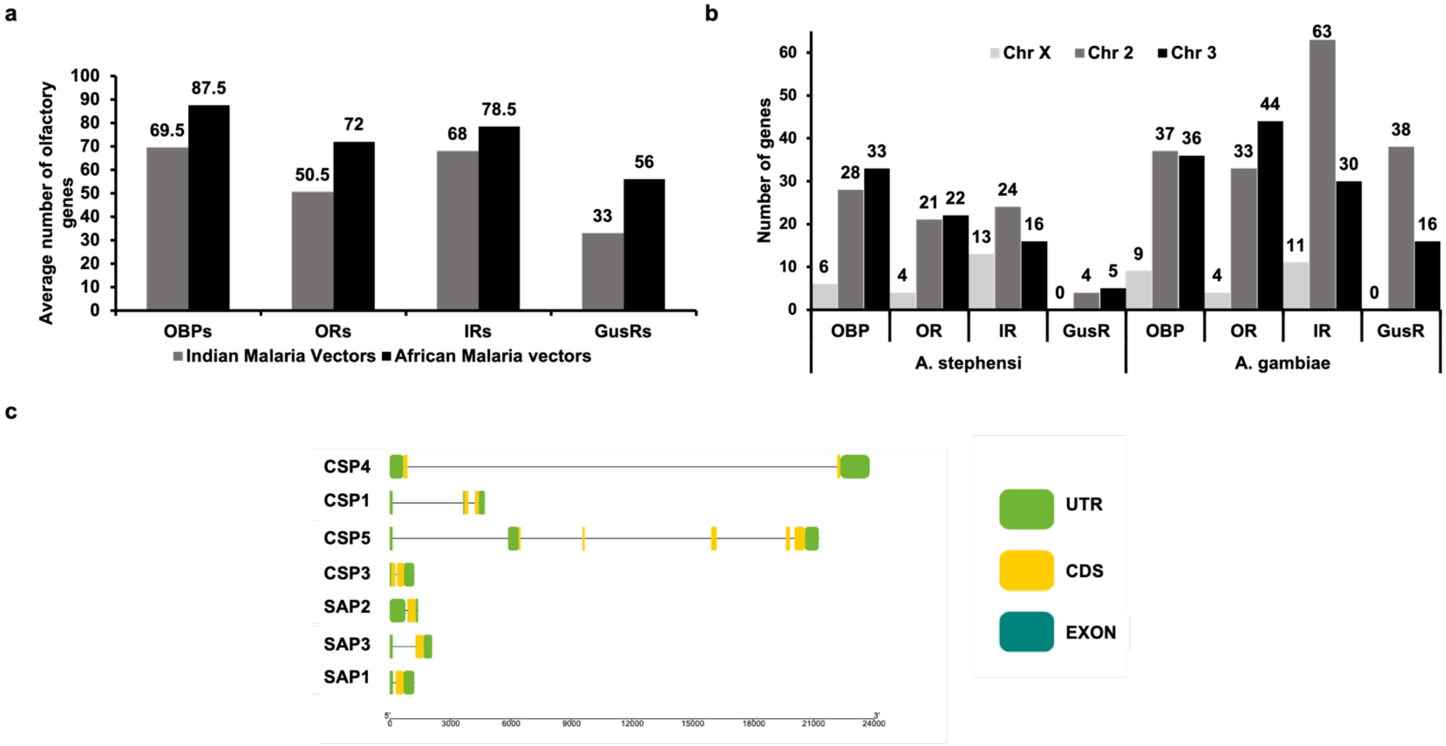

Figure S1. a) Average number of olfactory genes in Indian (*A. stephensi* & *A. culicifacies*) and African (*A. gambiae* & *A. funestus*) malaria vectors. b) Shows the chromosomal presence of olfactory genes in invasive *A. stephensi* and non-invasive *A. gambiae*. c) Detailed gene structure for CSPs and SAPs of *A. stephensi*.

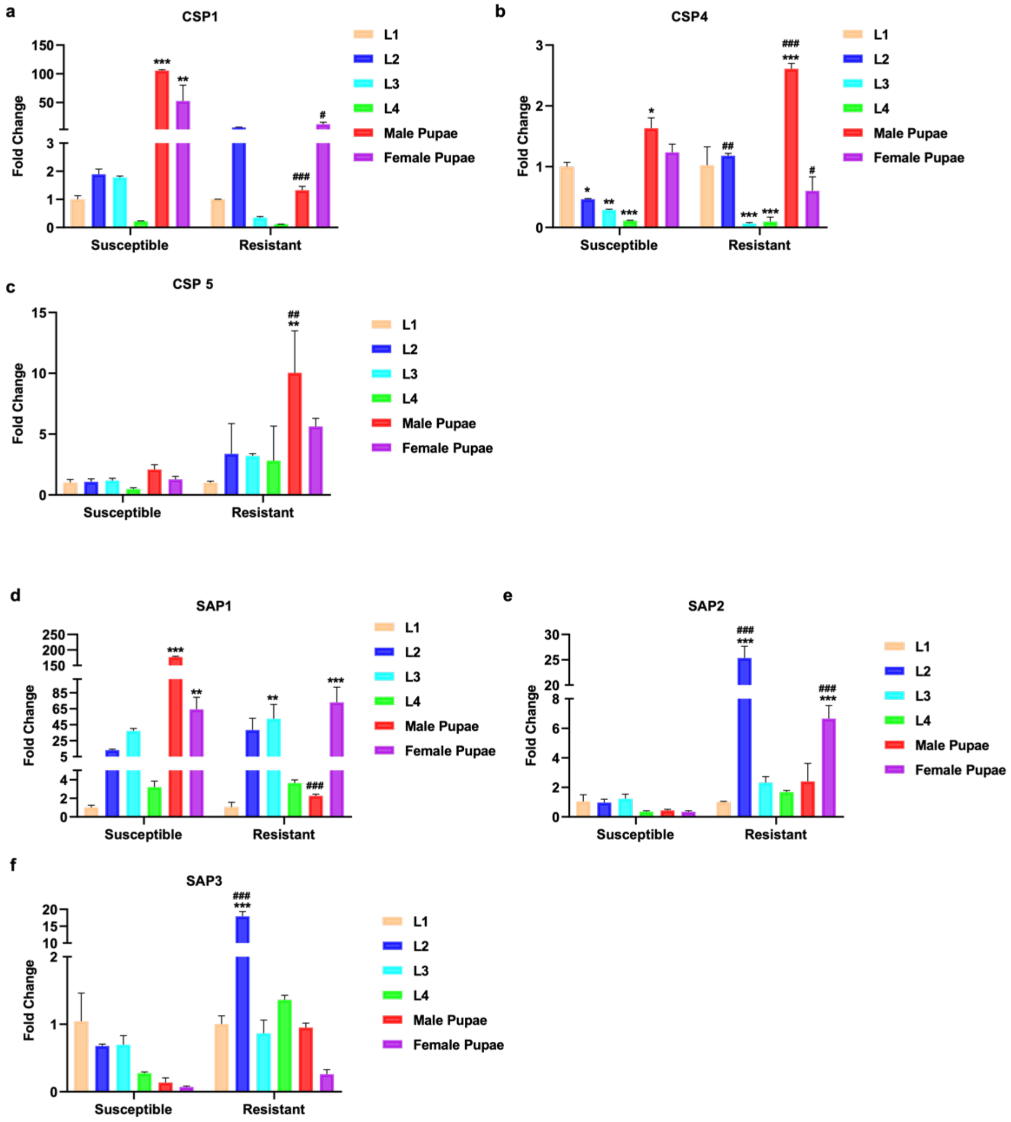

Figure S2. Expression of CSPs and SAPs in aquatic stages as determined by quantitative RT-PCR. L1 was used as a control for relative transcript expression and the $2^{-\Delta\Delta Ct}$ method was used for the analysis. The experiment was replicated two times with different biological cohorts of mosquitoes. Statistical significance was calculated by two-way ANOVA followed by tukey`s multiple comparison test using GraphPad Prism. Mean ± SD is shown in the figure. L1= first instar larvae, L2 = second instar larvae, L3= third instar larvae, and L4 = fourth instar larvae. * represents the statistical significance as compared to L1 stage of corresponding strain (*p<0.05, **p<0.01, and ***p<0.001). # represents the statistical significance compared to same stage from susceptible strain (#p<0.05, ##p<0.01, and ###p<0.001).

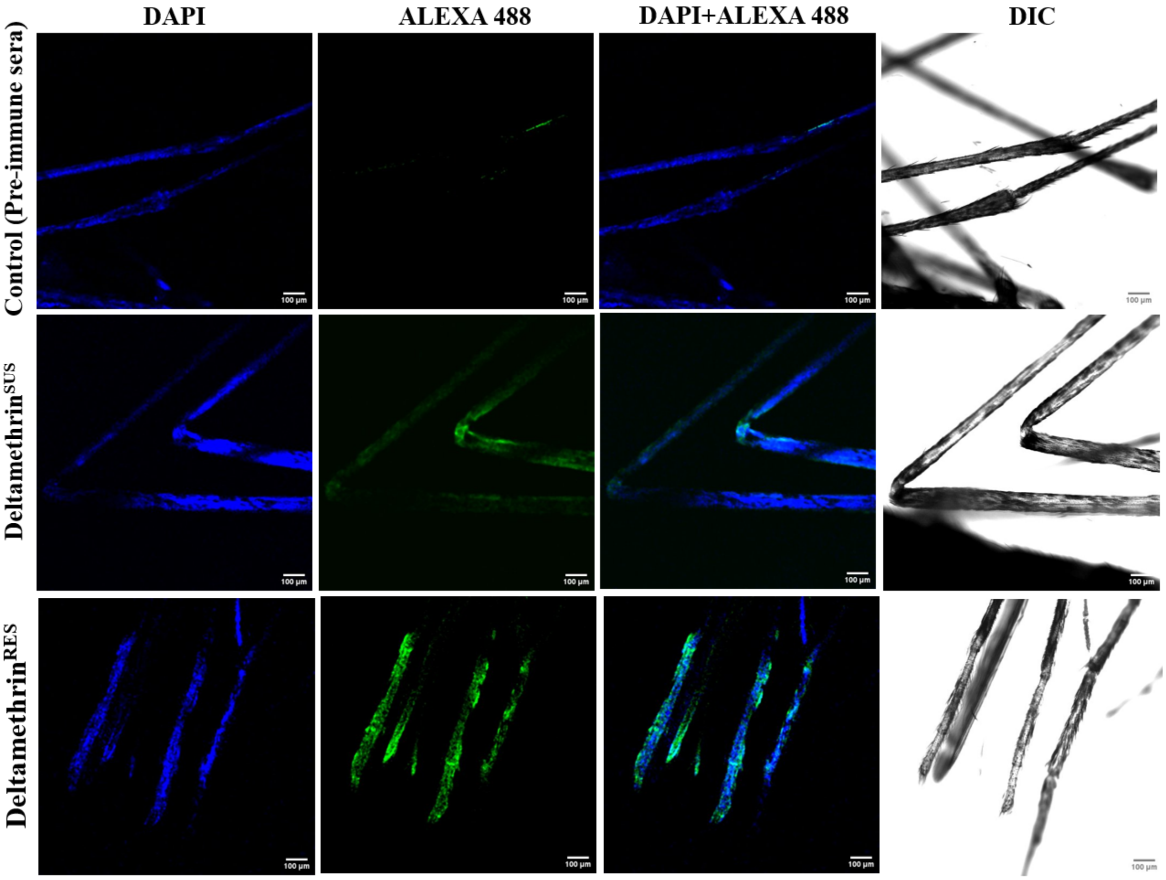

Figure S3. Immunofluorescence analysis of SAP2 expression in adult female legs of *Anopheles stephensi* from deltamethrin-susceptible and -resistant strains. Legs from third instar (L3) larvae of both susceptible and resistant strains were imaged following staining with DAPI (blue) and Alexa Fluor 488 (green; SAP2) at 10x magnification. Differential Interference Contrast (DIC) and corresponding fluorescent images are presented for both deltamethrin-susceptible (Deltamethrin SUS) and deltamethrin-resistant (Deltamethrin RES) strains. Control samples were incubated with mouse pre-bleed serum in place of the anti-SAP2 antibody (1:1000 dilution).

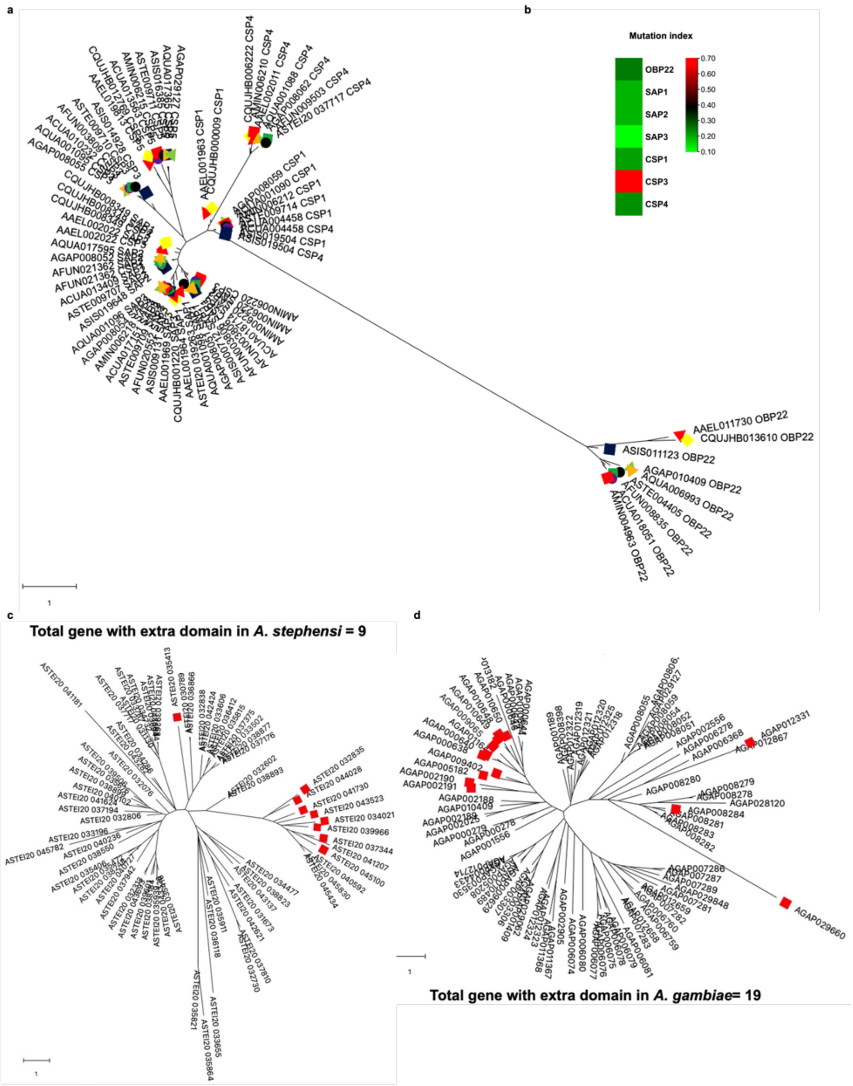

Figure S4. a) Maximum likelihood phylogenetic tree of the CSPs and SAPs amino acid sequences from 7 representative Anopheline, and 2 Culicine mosquito species with OBP22 and OBP1 as outlier. The ML tree was constructed using MEGA11, employing the JTT matrix model and 1000 bootstrap values. The tree displayed here represents the one with the highest log-likelihood. Branch lengths on the tree are scaled and measured in terms of substitutions per site. On the left-hand side panel, the comprehensive ML tree of OBP22 is shown with the color-coded selection scale to indicate positions undergoing positive or purifying selection at each amino acid location. Similarly, the right-hand side panel shows the comprehensive phylogenetic tree with a selection scale for SAP2. b) Figure showcases the heatmap of the mutation index for CSP, SAPs, and OBP22 in field-collected samples of *A. gambiae* obtained from the ag1000g database (https://www.malariagen.net/mosquito/ag1000g). The mutation index is calculated as the ratio of the total number of unique mutations to the total number of amino acids in the protein, corresponding to the overall length of the protein. ML phylogenetic tree based on amino acid sequences of OBP paralogs having domain duplication in c) *A. stephensi*, and d) *A. gambiae*. The genes with duplicated domains are shown with a red color marker on the phylogenetic tree. Vector base gene IDs are given in the phylogenetic tree and gene detail can be found in Table S1 & S2.

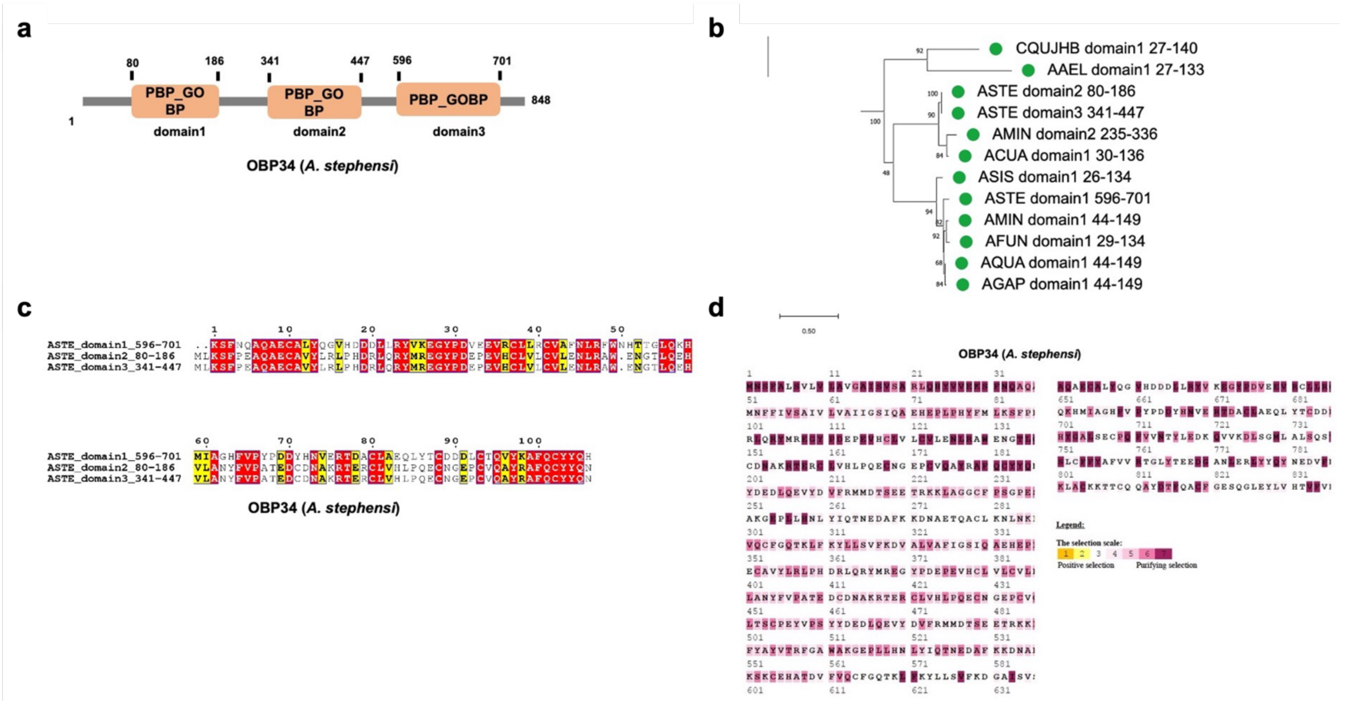

Figure S5. Domain duplication in OBP34 of *A. stephensi*. a) Duplicated domains position in the OBP34 protein. b) ML phylogenetic tree of OBP34 domains in 9 mosquito species (*A. stephensi, A. minimus, A. funestus, A quadriannulatus, A. gambiae, A. culicifacies, A. sinensis, Ae. aegypti,* and *C. quinquefasicatus*). c) Amino acid sequence alignment of three domains found in *A. stephensi* OBP34. The red color column represents the exact same amino acid in all aligned sequences and the yellow bar shows the similar charge amino acids. d) selection scale to indicate positions undergoing positive or purifying selection at each amino acid location.

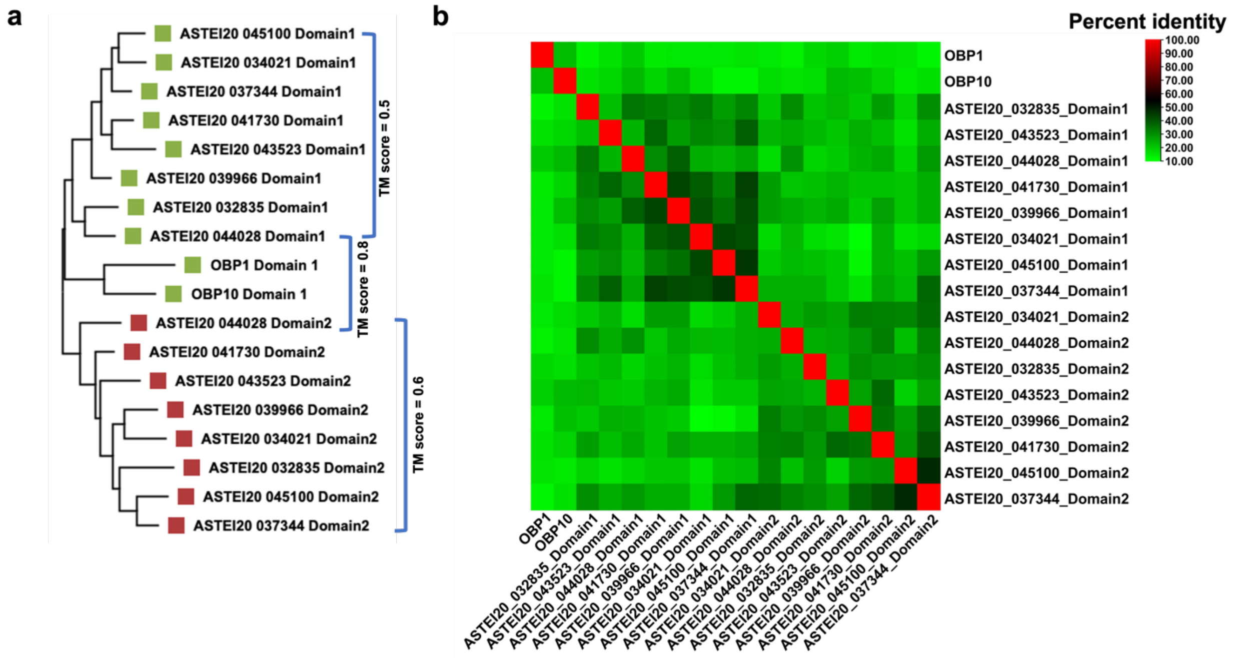

Figure S6. **a)** Maximum likelihood phylogenetic tree is shown, specifically focusing on OBPs (that exhibit domain duplication in *A. stephensi*. To identify the original domains within paralogs that have undergone domain duplication, OBP1, and OBP10 are used as reference points, as they possess a single PBP-GOBP domain. To provide additional insights into the structural similarity between domains, the tree incorporates the TM score. This score serves as a measure of the structural resemblance between two domains of the same gene, as well as between two domains from different genes. A TM score of 0 signifies structural dissimilarity, while a score of 1 indicates a high degree of structural similarity. **b)** Heatmap showing the percent identity of the amino acid sequences of *A. stephensi* OBPs paralogs having domain duplications cataloged in the current study.

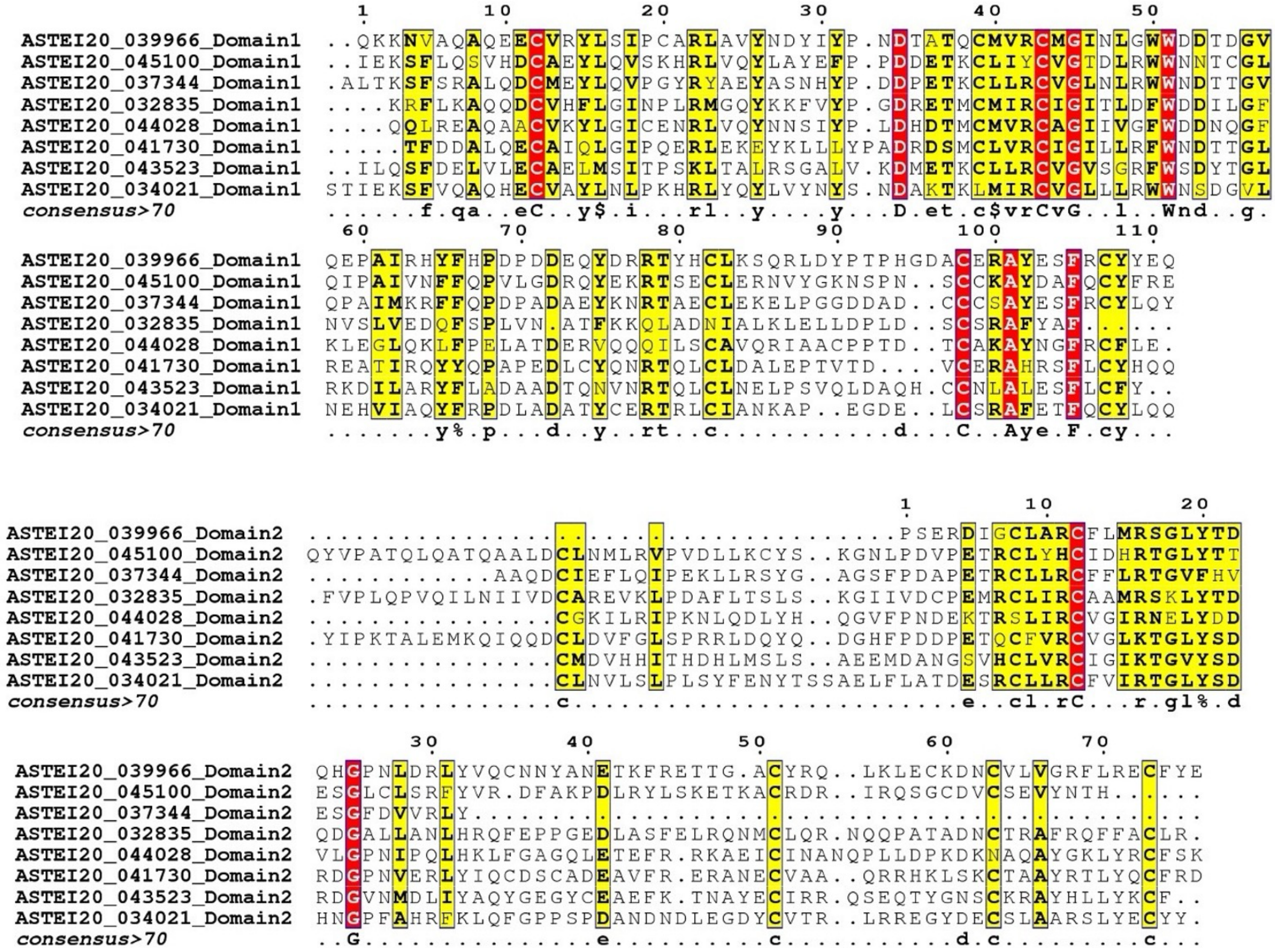

Figure S7. The amino acid sequence alignment of OBPs with domain duplication is displayed, with domain 1 and domain 2 shown separately. The red color column represents the exact same amino acid in all aligned sequences and the yellow bar shows the similar charge amino acids. The consensus sequence is given as the bottom line.

Supporting Tables:

Table

S1: List of crucial OBPs undergoing duplication and positive selection

	Classification	Conserved Domain	Doamin duplication	Seletcion	Ka/Ks ratio
**OBP69**	Plus C	PBP_GOBP	No	Purifying selection	0.055
**OBP68**	Classic	PBP_GOBP	Yes (*A. stephensi* (4) & *A. minimus* (4))	Positive selection at 9 sites; 105 N, 118 L, 234 S, 271 V ,281 G, 289 A, 291 Q, 294 Q, 477 Q	0.07
**OBP46**	Atypical	PBP_GOBP	No	Purifying selection	0.079
**OBP63**	Atypical	PBP_GOBP	No	Purifying selection	0.14808
**OBP21**	Classic	PBP_GOBP	No	Purifying selection	0.125
**SAP2**	minus C	OSD	No	Purifying selection	0.01473
**SAP3**	minus C	OSD	No	Purifying selection	0.05428
**CSP1**	minus C	OSD	No	Purifying selection	0.0312
**CSP4**	Dimer	OSD	No	Purifying selection	0.10769
**OBP34**	Classic	PBP_GOBP	Yes (*A. stephensi* (4) & *A. minimus* (4))	Purifying selection	0.103
**OBP9**	Classic	PBP_GOBP	No	Purifying selection	0.0319
**Male specific OBPs**					
	**Classification**	**Conserved Domain**	**Doamin duplication**	**Seletcion**	**Ka/Ks ratio**
SAP2	minus C	OSD	No	Positive selection at 2 sites ; 14V, 94 R	0.0738
SAP3	minus C	OSD	No		0.01473
CSP4-like	Dimer	OSD	No	Purifying selection	0.10796
OBP9	Classic	PBP_GOBP	No	Purifying selection	0.0319
CSP5	minus C	OSD	No	Purifying selection	0.12982
**Female specific OBPs**					
	**Classification**	**Conserved Domain**	**Doamin duplication**	**Seletcion**	**Ka/Ks ratio**
OBP54	minus C	PBP_GOBP	No	Purifying selection	0.17191
OBP7	Atypical	PBP_GOBP	No	Purifying selection	0.08592
OBP22	Classic	PBP_GOBP	No	Positive selection at one sitw ; 127 T	0.1566
OBP47	Atypical	PBP_GOBP	No	Purifying selection	0.0798
OBP1	Classic	PBP_GOBP	No	Purifying selection	0.04766
OBP5	Classic	PBP_GOBP	No	Purifying selection	0.06439

Table S2. Summarizing the number of olfactory genes cataloged in India as well as African major malaria vectors.

**Malaria vectors**	***Odorant Binding Proteins (OBPs)***	***Odorant Receptors (ORs)***	***Ionotropic Receptors (IRs)***	***Gustatory Receptors (GusRs)***
Anopheles stephensi	72 (4 *CSP* + 3 *SAP*)	47	55	9
Anopheles culicifacies	67	54	83	57
Anopheles gambiae	86	83	107 (60% on chromosome 2)	55
Anopheles funestus	89	61 (65% on chromosome 2)	50	57

Table S3. TPM values of identified important OBPs from VectorBase.

**Male-specific OBPs**		Remarks		Remarks
OBPs	***A. gambiae***		***A. stephensi***	
SAP2	TPM 367		TPM 1756	
SAP3	TPM 2615		TPM 2328	
Un/CSP4	TPM 23.2		TPM 27	
CSP4-like	TPM 23.2		TPM 51.63	
OBP9	TPM 621.98		TPM 1130	
CSP5	TPM 60.01		TPM 104.26	
**Female-specific OBPs**				
OBPs	***A. gambiae***		***A. stephensi***	
OBP54	TPM 93	FMP 93	TPM 14	Female 14; 36; Male 8.36
OBP7	TPM 19627	Female Antenna; TPM 19627	TPM 19.23	Female 19.23
OBP22	TPM 3187	Female Antenna; TPM 3189	TPM 62.86	Female 62.86
OBP47	TPM 9627	Female Antenna; TPM 9627	TPM 65	Female 65
OBP1	TPM 47319	Female Antenna; 470319	TPM 46	Female 46; Male 22
OBP54	TPM 14627	FA 14627	TPM 6.91	6.91
**Aquatic stages**				
OBPs	***A. gambiae***		***A. stephensi***	
OBP69	TPM 0.01	VLE	TPM 73	TPM 73 pupa
OBP68	TPM 170	TPM L3170	TPM 60	TPM 60
OBP46	TPM 423	48h after lying eggs; TPM 423	TPM 151.4	TPM 151.25
OPB63	TPM 113.87	48h after lying eggs; TPM 113.87	TPM 71.75	TPM 71.75
OBP21	TPM 174	L2-L3; 174	TPM 77	TPM 77 pupa
SAP2	TPM 462	48h after egg hatching 462; Male sp.	TPM 148	TPM 148
SAP3	TPM 3975	Female larve; 3975	TPM 2970.9	TPM 2970
CSP1	TPM 1452	5hrs after lying eggs; 1452	TPM 1016	TPM 1016; 4-8 after embryo emergrnce
CSP4	TPM 77	24 hr after laying eggs; 77	TPM 17.36	TPM 17.36; pupa
OBP34	TPM 0.01	VLE	TPM 11	TPM 4; pupa
OBP9	TPM 4869	Larve, 24 h after lying eggs TPM 4869	TPM 4021	TPM 4021 pupa

Table S4. List of primers used to amplify CSPs and SAPs for relative expression by qRT-PCR in *A. stephensi*.

S. No.	Primer Name	Sequence
1	*AnsSAP3_1F*	5' AGACGGACTGTGCCAAGTG 3'
2	*AnsSAP3_1R*	5' TGTTCTCCGGGTCGTACTTC 3'
3	*AnsSAP2_1F*	5' AGTACACCACCAAGTACGACG 3'
4	*AnsSAP2_1R*	5' CTTCAGCTCGTTACCGTCCG 3'
5	*AnsSAP1_1F*	5' TAACGAGCTGAAGAAGATCCTGC 3'
6	*AnsSAP1_1R*	5' TTCCAGCTTGATGCCCTCCTTC 3'
7	*AnsCSP5_1F*	5' CATTGCGTACCAAGTGTGCC 3'
8	*AnsCSP5_1R*	5' TATTCCTCGAACCGGCGATG 3'
9	*AnsCSP4_1F*	5' ATGTGCGTCCTGGAGAAGAG 3'
10	*AnsCSP4_1R*	5' GGAAGTTCGTCAGCTTCTGG 3'
11	*AnsCSP1_1F*	5' CTTCCCGATGCGCTGAAAAC 3'
12	*AnsCSP1_1R*	5' TCCATTCCTGTGGCTTACGG 3'
13	*Actin Fw*	5' TGCGTGACATCAAGGAGAAG 3'
14	*Actin Rev*	5' GATTCCATACCCAGGAACGA 3'

Table S5. WHO recommended susceptibility assay for *A. stephensi*

**Mosquitoes**	**Control % Mortality (n)**	**Test % Mortality (n)**	**Status**
A/S Susceptible	0% (50)	100% (100)	Deltamethrin susceptible strain
A/S Resistant	0% (50)	21% (100)	Deltamethrin Resistant strain

n= Number of mosquitoes used for the assay in multiple batches

***Table SX1. Showing detailed information about the identified olfactory gene (*OBPs*, *ORs*, *IRs*, and *GusRs*) in Indian malaria vectors *A. stephensi* and *A. culicifacies*. (*Additional Excel file attached)**

****Table SX2. Showing detailed information about the identified olfactory gene (*OBPs*, *ORs*, *IRs*, and *GusRs*) in African malaria vectors *A. gambiae* and *A. funestus*. (**Additional Excel file attached)**
